# (*E*)-2-({2-[(*E*)-(Hy­droxy­imino)­meth­yl]phen­oxy}meth­yl)-3-phenyl­acrylonitrile

**DOI:** 10.1107/S1600536812003923

**Published:** 2012-02-04

**Authors:** Suresh Govindan, Sabari Vijayakumar, Srinivasan Jayakumar, Bakthadoss Mannickam, Aravindhan Sanmargam

**Affiliations:** a Department of Physics, Presidency College, Chennai 600 005, India; b Department of Organic Chemistry, University of Madras, Chennai 600 025, India

## Abstract

In the title compound, C_17_H_14_N_2_O_2_, the hy­droxy­ethanimine group adopts an anti­periplanar conformation. In the crystal, mol­ecules are linked by O—H⋯N hydrogen bonds, forming zigzag chains running along the *c* axis.

## Related literature

For the structures of other acrylate derivatives, see: Zhang *et al.* (2009 ▶); Wang *et al.* (2011 ▶); SakthiMurugesan *et al.* (2011 ▶); Govindan *et al.* (2011 ▶). For the use of oxime ligands in coordination chemistry, see: Chaudhuri (2003 ▶). For the biological activity of caffeic acids, see: Hwang *et al.* (2001 ▶); Altug *et al.* (2008 ▶); Ates *et al.* (2006 ▶); Atik *et al.* (2006 ▶); Padinchare *et al.* (2001 ▶).
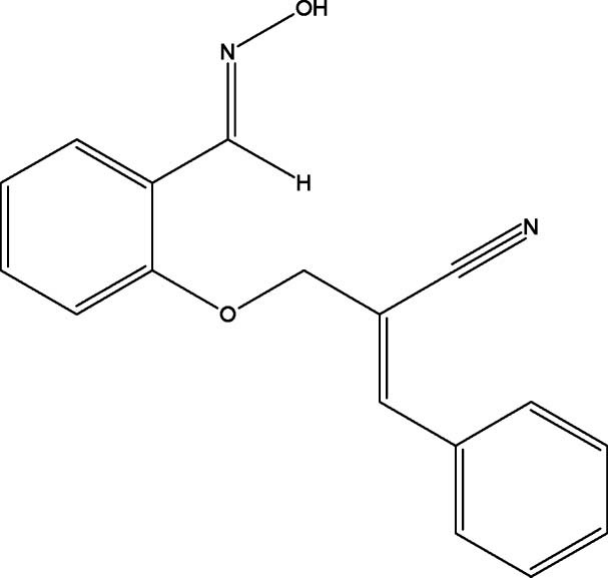


## Experimental

### 

#### Crystal data

C_17_H_14_N_2_O_2_
*M* *_r_* = 278.30
Monoclinic, 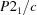

*a* = 15.8867 (5) Å
*b* = 6.2381 (2) Å
*c* = 15.1874 (4) Å
β = 107.199 (2)°
*V* = 1437.81 (7) Å^3^
*Z* = 4
Mo *K*α radiation
μ = 0.09 mm^−1^
*T* = 293 K
0.2 × 0.2 × 0.2 mm

#### Data collection

Oxford Diffraction Xcalibur-S diffractometer
Absorption correction: multi-scan (*CrysAlis PRO*; Oxford Diffraction, 2009 ▶) *T*_min_ = 0.980, *T*_max_ = 0.990
19516 measured reflections
4490 independent reflections
2774 reflections with *I* > 2σ(*I*)
*R*_int_ = 0.031

#### Refinement

*R*[*F* ^2^ > 2σ(*F* ^2^)] = 0.049
*wR*(*F* ^2^) = 0.131
*S* = 0.99
4490 reflections
191 parameters
H-atom parameters constrained
Δρ_max_ = 0.19 e Å^−3^
Δρ_min_ = −0.21 e Å^−3^

### 

Data collection: *CrysAlis PRO* (Oxford Diffraction, 2009 ▶); cell refinement: *CrysAlis PRO*; data reduction: *CrysAlis PRO*; program(s) used to solve structure: *SHELXS97* (Sheldrick, 2008 ▶); program(s) used to refine structure: *SHELXL97* (Sheldrick, 2008 ▶); molecular graphics: *ORTEP-3 for Windows* (Farrugia, 1997 ▶); software used to prepare material for publication: *PLATON* (Spek, 2009 ▶).

## Supplementary Material

Crystal structure: contains datablock(s) I, global. DOI: 10.1107/S1600536812003923/bt5765sup1.cif

Structure factors: contains datablock(s) I. DOI: 10.1107/S1600536812003923/bt5765Isup2.hkl

Supplementary material file. DOI: 10.1107/S1600536812003923/bt5765Isup3.cml

Additional supplementary materials:  crystallographic information; 3D view; checkCIF report

## Figures and Tables

**Table 1:** Hydrogen-bond geometry (Å, °)

*D*—H⋯*A*	*D*—H	H⋯*A*	*D*⋯*A*	*D*—H⋯*A*
O1—H1*A*⋯N2^i^	0.82	2.10	2.9187 (17)	178

Symmetry code: (i) 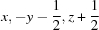.

**Fig. 1.**
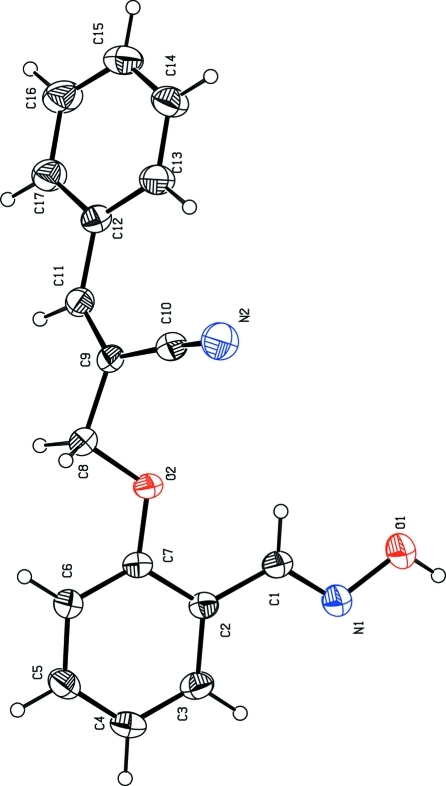
The molecular structure of the title compound, showing 30% probability displacement ellipsoids for non-H atoms.

**Fig. 2.**
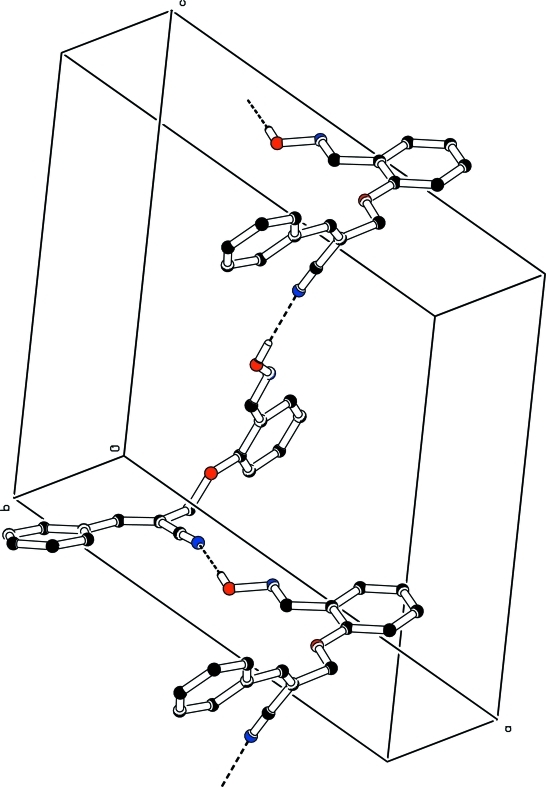
A view of the crystal packing. H atoms not involved in hydrogen bonding (dashed lines) have been omitted for clarity.

## supplementary crystallographic information

### Comment

Recently, 2-cyanoacrylates have been extensively used as agrochemicals because of their unique mechanism of action and good environmental profiles (Zhang *et al.*, 2009). Oximes are a classical type of chelating ligands which are widely used in coordination and analytical chemistry (Chaudhuri, 2003). Some naturally occurring caffeic acids and their esters attract much attention in biology and medicine (Hwang *et al.*, 2001; Altug *et al.*, 2008). These compounds show antiviral, antibacterial, vasoactive, antiatherogenic, antiproliferative, antioxidant and antiinflammatory properties (Atik *et al.*, 2006; Padinchare *et al.*, 2001; Ates *et al.*, 2006). Against this background, and in order to obtain detailed information on molecular conformations in the solid state, an X-ray study of the title compound was carried out and the results are presented here. X-Ray analysis confirms the molecular structure and atom connectivity as illustrated in Fig. 1. The oxime group has the C=N bond in an E configuration. The hydroxy ethanimine group is essentially coplanar with the ring to which it is attached. The crystal packing is stabilized by an O—H···N hydrogen bond(Fig. 2).

### Experimental

To a stirred solution of (*E*)-2-((2-formylphenoxy)methyl)-3-phenylacrylonitrile (4 mmol) in 10 ml of EtOH/H2O mixture (1:1) was added NH2OH.HCl (6 mmol) in the presence of 50% NaOH at room temperature. Then the reaction mixture was allowed to stir at room temperature for 1.5 h. After completion of the reaction, solvent was removed and the crude mass was diluted with water (15 ml) and extracted with ethyl acetate (3 τimes 15 ml). The combined organic layer was washed with brine (2 τimes 10 ml) and dried over anhydrous Na2SO4 and then evaporated under reduced pressure to obtain (*E*)-2-((2-((*E*)-(Hydroxyimino)methyl)phenoxy)methyl)-3-phenylacrylonitrile as a colourless solid.

### Refinement

H atoms were positioned at calculated positions and refined using a riding model with O-H=0.82Å, C_aromatic_-H = 0.93Å and C_methylene_-H= 0.97Å and U(H) set to 1.2U_eq_(C) or 1.5U_eq_(O).

### Crystal data

**Table:**

C_17_H_14_N_2_O_2_	*F*(000) = 584
*M**_r_* = 278.30	*D*_x_ = 1.286 Mg m^−^^3^
Monoclinic, *P*2_1_/*c*	Mo *K*α radiation, λ = 0.71073 Å
Hall symbol: -P 2ybc	Cell parameters from 8725 reflections
*a* = 15.8867 (5) Å	θ = 2.8–29.1°
*b* = 6.2381 (2) Å	µ = 0.09 mm^−^^1^
*c* = 15.1874 (4) Å	*T* = 293 K
β = 107.199 (2)°	Monoclinic, colourless
*V* = 1437.81 (7) Å^3^	0.2 × 0.2 × 0.2 mm
*Z* = 4	

### Data collection

**Table:**

Oxford Diffraction Xcalibur-S diffractometer	4490 independent reflections
Radiation source: fine-focus sealed tube	2774 reflections with *I* > 2σ(*I*)
Graphite monochromator	*R*_int_ = 0.031
Detector resolution: 15.9948 pixels mm^-1^	θ_max_ = 31.4°, θ_min_ = 2.7°
ω scans	*h* = −20→23
Absorption correction: multi-scan (*CrysAlis PRO*; Oxford Diffraction, 2009)	*k* = −9→9
*T*_min_ = 0.980, *T*_max_ = 0.990	*l* = −22→22
19516 measured reflections	

### Refinement

**Table:**

Refinement on *F*^2^	Primary atom site location: structure-invariant direct methods
Least-squares matrix: full	Secondary atom site location: difference Fourier map
*R*[*F*^2^ > 2σ(*F*^2^)] = 0.049	Hydrogen site location: inferred from neighbouring sites
*wR*(*F*^2^) = 0.131	H-atom parameters constrained
*S* = 0.99	*w* = 1/[σ^2^(*F*_o_^2^) + (0.0521*P*)^2^ + 0.226*P*] where *P* = (*F*_o_^2^ + 2*F*_c_^2^)/3
4490 reflections	(Δ/σ)_max_ < 0.001
191 parameters	Δρ_max_ = 0.19 e Å^−^^3^
0 restraints	Δρ_min_ = −0.21 e Å^−^^3^

### Special details

**Table:**

Geometry. All e.s.d.'s (except the e.s.d. in the dihedral angle between two l.s. planes) are estimated using the full covariance matrix. The cell e.s.d.'s are taken into account individually in the estimation of e.s.d.'s in distances, angles and torsion angles; correlations between e.s.d.'s in cell parameters are only used when they are defined by crystal symmetry. An approximate (isotropic) treatment of cell e.s.d.'s is used for estimating e.s.d.'s involving l.s. planes.
Refinement. Refinement of *F*^2^ against ALL reflections. The weighted *R*-factor *wR* and goodness of fit *S* are based on *F*^2^, conventional *R*-factors *R* are based on *F*, with *F* set to zero for negative *F*^2^. The threshold expression of *F*^2^ > σ(*F*^2^) is used only for calculating *R*-factors(gt) *etc*. and is not relevant to the choice of reflections for refinement. *R*-factors based on *F*^2^ are statistically about twice as large as those based on *F*, and *R*- factors based on ALL data will be even larger.

### Fractional atomic coordinates and isotropic or equivalent isotropic displacement parameters (Å^2^)

**Table:**

	*x*	*y*	*z*	*U*_iso_*/*U*_eq_	
C1	0.30906 (9)	0.0194 (2)	0.29768 (9)	0.0471 (3)	
H1	0.2662	0.0231	0.2407	0.057*	
C2	0.37843 (8)	0.1812 (2)	0.31995 (8)	0.0405 (3)	
C3	0.44575 (9)	0.1763 (2)	0.40269 (9)	0.0500 (3)	
H3	0.4456	0.0697	0.4455	0.060*	
C4	0.51256 (9)	0.3247 (2)	0.42297 (9)	0.0525 (3)	
H4	0.5574	0.3176	0.4784	0.063*	
C5	0.51234 (9)	0.4837 (2)	0.36050 (10)	0.0542 (4)	
H5	0.5574	0.5849	0.3740	0.065*	
C6	0.44610 (9)	0.4954 (2)	0.27781 (9)	0.0488 (3)	
H6	0.4464	0.6041	0.2360	0.059*	
C7	0.37943 (8)	0.3449 (2)	0.25758 (8)	0.0399 (3)	
C8	0.30879 (9)	0.5024 (2)	0.11052 (9)	0.0474 (3)	
H8A	0.3612	0.4952	0.0904	0.057*	
H8B	0.3058	0.6435	0.1363	0.057*	
C9	0.22813 (8)	0.4609 (2)	0.03129 (8)	0.0411 (3)	
C10	0.22894 (9)	0.2607 (2)	−0.01427 (9)	0.0486 (3)	
C11	0.16197 (9)	0.6014 (2)	0.00727 (8)	0.0444 (3)	
H11	0.1718	0.7251	0.0431	0.053*	
C12	0.07740 (9)	0.5990 (2)	−0.06398 (8)	0.0435 (3)	
C13	0.04331 (10)	0.4260 (2)	−0.12169 (10)	0.0577 (4)	
H13	0.0768	0.3021	−0.1177	0.069*	
C14	−0.03912 (10)	0.4363 (3)	−0.18424 (10)	0.0629 (4)	
H14	−0.0607	0.3196	−0.2223	0.075*	
C15	−0.08983 (10)	0.6164 (3)	−0.19123 (11)	0.0633 (4)	
H15	−0.1460	0.6212	−0.2330	0.076*	
C16	−0.05728 (11)	0.7892 (3)	−0.13634 (12)	0.0694 (5)	
H16	−0.0911	0.9128	−0.1414	0.083*	
C17	0.02527 (10)	0.7807 (2)	−0.07360 (10)	0.0577 (4)	
H17	0.0466	0.8996	−0.0368	0.069*	
N1	0.30657 (8)	−0.12586 (19)	0.35497 (8)	0.0527 (3)	
N2	0.23413 (9)	0.0992 (2)	−0.04726 (10)	0.0728 (4)	
O1	0.23639 (8)	−0.26430 (19)	0.31926 (8)	0.0716 (3)	
H1A	0.2369	−0.3587	0.3570	0.107*	
O2	0.31108 (6)	0.34087 (15)	0.17729 (6)	0.0487 (2)	

### Atomic displacement parameters (Å^2^)

**Table:**

	*U*^11^	*U*^22^	*U*^33^	*U*^12^	*U*^13^	*U*^23^
C1	0.0432 (7)	0.0502 (8)	0.0433 (7)	0.0001 (6)	0.0055 (6)	0.0077 (6)
C2	0.0369 (6)	0.0437 (7)	0.0396 (6)	0.0029 (5)	0.0093 (5)	0.0023 (5)
C3	0.0488 (8)	0.0581 (8)	0.0397 (7)	0.0044 (7)	0.0079 (6)	0.0084 (6)
C4	0.0455 (8)	0.0654 (9)	0.0387 (7)	0.0008 (7)	0.0004 (6)	−0.0043 (6)
C5	0.0488 (8)	0.0567 (9)	0.0514 (8)	−0.0101 (7)	0.0061 (6)	−0.0076 (7)
C6	0.0479 (8)	0.0474 (8)	0.0470 (7)	−0.0046 (6)	0.0075 (6)	0.0040 (6)
C7	0.0359 (6)	0.0441 (7)	0.0372 (6)	0.0032 (5)	0.0070 (5)	0.0013 (5)
C8	0.0481 (8)	0.0434 (7)	0.0456 (7)	−0.0020 (6)	0.0060 (6)	0.0092 (6)
C9	0.0460 (7)	0.0386 (6)	0.0367 (6)	−0.0009 (5)	0.0090 (5)	0.0048 (5)
C10	0.0443 (8)	0.0476 (8)	0.0497 (7)	0.0045 (6)	0.0076 (6)	0.0034 (6)
C11	0.0524 (8)	0.0397 (7)	0.0387 (6)	0.0005 (6)	0.0096 (6)	−0.0009 (5)
C12	0.0453 (7)	0.0462 (7)	0.0377 (6)	0.0033 (6)	0.0104 (5)	0.0028 (5)
C13	0.0551 (9)	0.0542 (9)	0.0547 (8)	0.0079 (7)	0.0023 (7)	−0.0070 (7)
C14	0.0565 (10)	0.0695 (10)	0.0541 (9)	−0.0032 (8)	0.0030 (7)	−0.0095 (7)
C15	0.0449 (8)	0.0833 (12)	0.0551 (9)	0.0031 (8)	0.0048 (7)	0.0054 (8)
C16	0.0536 (10)	0.0700 (11)	0.0773 (11)	0.0192 (8)	0.0080 (8)	0.0012 (9)
C17	0.0551 (9)	0.0527 (9)	0.0602 (9)	0.0091 (7)	0.0093 (7)	−0.0048 (7)
N1	0.0521 (7)	0.0517 (7)	0.0522 (7)	−0.0081 (5)	0.0119 (5)	0.0032 (5)
N2	0.0731 (10)	0.0553 (8)	0.0837 (10)	0.0116 (7)	0.0136 (8)	−0.0131 (7)
O1	0.0701 (8)	0.0631 (7)	0.0727 (7)	−0.0239 (6)	0.0074 (6)	0.0106 (6)
O2	0.0427 (5)	0.0525 (5)	0.0421 (5)	−0.0062 (4)	−0.0009 (4)	0.0133 (4)

### Geometric parameters (Å, º)

**Table:**

C1—N1	1.2649 (16)	C9—C11	1.3339 (18)
C1—C2	1.4583 (18)	C9—C10	1.4296 (19)
C1—H1	0.9300	C10—N2	1.1392 (17)
C2—C3	1.3889 (18)	C11—C12	1.4548 (18)
C2—C7	1.3962 (17)	C11—H11	0.9300
C3—C4	1.373 (2)	C12—C17	1.3857 (19)
C3—H3	0.9300	C12—C13	1.3951 (19)
C4—C5	1.372 (2)	C13—C14	1.373 (2)
C4—H4	0.9300	C13—H13	0.9300
C5—C6	1.3814 (19)	C14—C15	1.368 (2)
C5—H5	0.9300	C14—H14	0.9300
C6—C7	1.3804 (18)	C15—C16	1.367 (2)
C6—H6	0.9300	C15—H15	0.9300
C7—O2	1.3725 (14)	C16—C17	1.375 (2)
C8—O2	1.4225 (14)	C16—H16	0.9300
C8—C9	1.4983 (18)	C17—H17	0.9300
C8—H8A	0.9700	N1—O1	1.3876 (15)
C8—H8B	0.9700	O1—H1A	0.8200
			
N1—C1—C2	120.76 (12)	C11—C9—C8	121.58 (12)
N1—C1—H1	119.6	C10—C9—C8	114.31 (11)
C2—C1—H1	119.6	N2—C10—C9	176.18 (16)
C3—C2—C7	118.02 (12)	C9—C11—C12	132.56 (12)
C3—C2—C1	121.43 (12)	C9—C11—H11	113.7
C7—C2—C1	120.55 (11)	C12—C11—H11	113.7
C4—C3—C2	121.70 (13)	C17—C12—C13	117.24 (13)
C4—C3—H3	119.2	C17—C12—C11	117.49 (12)
C2—C3—H3	119.2	C13—C12—C11	125.23 (12)
C5—C4—C3	119.26 (12)	C14—C13—C12	120.79 (14)
C5—C4—H4	120.4	C14—C13—H13	119.6
C3—C4—H4	120.4	C12—C13—H13	119.6
C4—C5—C6	120.82 (13)	C15—C14—C13	120.76 (15)
C4—C5—H5	119.6	C15—C14—H14	119.6
C6—C5—H5	119.6	C13—C14—H14	119.6
C7—C6—C5	119.64 (12)	C16—C15—C14	119.48 (14)
C7—C6—H6	120.2	C16—C15—H15	120.3
C5—C6—H6	120.2	C14—C15—H15	120.3
O2—C7—C6	124.30 (11)	C15—C16—C17	120.22 (15)
O2—C7—C2	115.14 (11)	C15—C16—H16	119.9
C6—C7—C2	120.56 (11)	C17—C16—H16	119.9
O2—C8—C9	106.60 (10)	C16—C17—C12	121.48 (15)
O2—C8—H8A	110.4	C16—C17—H17	119.3
C9—C8—H8A	110.4	C12—C17—H17	119.3
O2—C8—H8B	110.4	C1—N1—O1	111.22 (11)
C9—C8—H8B	110.4	N1—O1—H1A	109.5
H8A—C8—H8B	108.6	C7—O2—C8	117.86 (9)
C11—C9—C10	124.11 (12)		
			
N1—C1—C2—C3	2.7 (2)	C8—C9—C11—C12	−178.61 (12)
N1—C1—C2—C7	−178.10 (13)	C9—C11—C12—C17	−176.32 (13)
C7—C2—C3—C4	−1.05 (19)	C9—C11—C12—C13	5.7 (2)
C1—C2—C3—C4	178.19 (12)	C17—C12—C13—C14	−0.9 (2)
C2—C3—C4—C5	0.8 (2)	C11—C12—C13—C14	177.09 (14)
C3—C4—C5—C6	−0.2 (2)	C12—C13—C14—C15	−0.2 (2)
C4—C5—C6—C7	−0.2 (2)	C13—C14—C15—C16	1.1 (3)
C5—C6—C7—O2	−179.43 (12)	C14—C15—C16—C17	−1.0 (3)
C5—C6—C7—C2	0.0 (2)	C15—C16—C17—C12	−0.1 (3)
C3—C2—C7—O2	−179.90 (11)	C13—C12—C17—C16	1.1 (2)
C1—C2—C7—O2	0.86 (16)	C11—C12—C17—C16	−177.09 (14)
C3—C2—C7—C6	0.63 (18)	C2—C1—N1—O1	−178.97 (12)
C1—C2—C7—C6	−178.62 (12)	C6—C7—O2—C8	0.00 (18)
O2—C8—C9—C11	116.39 (13)	C2—C7—O2—C8	−179.45 (11)
O2—C8—C9—C10	−63.22 (14)	C9—C8—O2—C7	−179.66 (10)
C10—C9—C11—C12	1.0 (2)		

### Hydrogen-bond geometry (Å, º)

**Table:**

*D*—H···*A*	*D*—H	H···*A*	*D*···*A*	*D*—H···*A*
O1—H1*A*···N2^i^	0.82	2.10	2.9187 (17)	178

Symmetry code: (i) *x*, −*y*−1/2, *z*+1/2.
